# Metabolic Reprograming of Mononuclear Phagocytes in Progressive Multiple Sclerosis

**DOI:** 10.3389/fimmu.2015.00106

**Published:** 2015-03-11

**Authors:** Gillian Margaret Tannahill, Nunzio Iraci, Edoardo Gaude, Christian Frezza, Stefano Pluchino

**Affiliations:** ^1^Department of Clinical Neurosciences, NIHR Biomedical Research Centre, University of Cambridge, Cambridge, UK; ^2^Wellcome Trust-Medical Research Council (MRC) Stem Cell Institute, Cambridge, UK; ^3^MRC Cancer Unit, Hutchison/MRC Research Centre, University of Cambridge, Cambridge, UK

**Keywords:** immune metabolism, macrophages, microglia, Warburg effect, multiple sclerosis, EAE, mitochondria

## Abstract

Multiple sclerosis (MS) is an inflammatory and demyelinating disease of the central nervous system (CNS). Accumulation of brain damage in progressive MS is partly the result of mononuclear phagocytes (MPs) attacking myelin sheaths in the CNS. Although there is no cure yet for MS, significant advances have been made in the development of disease modifying agents. Unfortunately, most of these drugs fail to reverse established neurological deficits and can have adverse effects. Recent evidence suggests that MPs polarization is accompanied by profound metabolic changes, whereby pro-inflammatory MPs (M1) switch toward glycolysis, whereas anti-inflammatory MPs (M2) become more oxidative. It is therefore possible that reprograming MPs metabolism could affect their function and repress immune cell activation. This *mini review* describes the metabolic changes underpinning macrophages polarization and anticipates how metabolic re-education of MPs could be used for the treatment of MS.

**Key points:**

- Inflammation in progressive MS is mediated primarily by MPs.
- Cell metabolism regulates the function of MPs.
- DMAs can re-educate the metabolism of MPs to promote healing.

## Introduction

Multiple sclerosis (MS) is an inflammatory disease of the central nervous system (CNS) in which perivascular infiltration of self-reactive T lymphocytes leads to demyelination (both primary and secondary) and axonal damage. Inflammation is an early and transient event in MS and remyelination occurs afterwards (1). The early stages of the disease are characterized by episodes of neurological dysfunction that usually recover. Over time, the pathological features of MS become dominated by widespread microglial activation associated with extensive and chronic neurodegeneration, which associates with progressive accumulation of disability (2).

Current immune modulatory treatments are effective at reducing T-cell-mediated damage early in disease (3). However, most of these therapeutic strategies have failed to work in patients with progressive MS, where uncontrolled activation of mononuclear phagocytes (MPs) takes place in the chronically inflamed CNS (4–6).

Mononuclear phagocytes, such as microglia and macrophages, are present in all tissues where they have a range of homeostatic functions including the removal of apoptotic cells and cell debris (7). Although functionally similar, microglia and macrophages are ontogenetically distinct populations (8–11). Microglia, the primary MPs in the CNS, are derived from the yolk-sac blood islands and migrate to the neuroepithelium during early development (7, 12, 13). Microglia interact with neural progenitor cells to regulate both structural and functional responses in the CNS during development, homeostasis, and disease (14, 15). Macrophages are derived from hematopoietic stem cells in the bone marrow that differentiate into peripheral blood monocytes (16). Macrophages are critical for innate immune defense and also control organ homeostasis in a tissue-specific manner. In non-parenchymal areas of the CNS, macrophages and microglia survey for tissue injury and infection (17).

Mononuclear phagocytes are phenotypically classified as classically activated (M1-like; pro-inflammatory) or alternatively activated (M2-like; anti-inflammatory) cells. This paradigm should not be over-interpreted, as it is not a rigid classification. M1-like MPs produce neurotoxic molecules, pro-inflammatory cytokines, and chemokines and present self-antigens to attract cytotoxic CD8^+^ T cells (18, 19), whereas M2-like MPs are regenerative cells that secrete growth and neurotropic factors (20, 21). MPs polarization is governed by *intrinsic* (22) and *extrinsic* factors, and even differentiated macrophages can be reprogramed when transferred into a new microenvironment (23).

Accumulation and activation of MPs in the CNS is thought to be a crucial step in the pathological cascade of MS, which frequently culminates in irreversible injury to myelin and axons (24). Therefore, MS therapies that steer MPs toward a reparative, rather than pro-inflammatory, phenotype are now emerging as ideal approaches to promote tissue healing without disrupting MPs functions.

This *mini review* describes the metabolic changes underpinning macrophages polarization and anticipates how metabolic re-education of MPs could be used for the treatment of MS.

## Progressive MS and MPs

A balanced response between the M1- and M2-like phenotype is necessary for tissue homeostasis, and in MS and its animal model experimental autoimmune encephalomyelitis (EAE), this balance is disturbed. The fact that the MS *per se* exists in the relapsing/remitting type points to the M1/M2 dynamics as potentially relevant for this disease (25–27).

By expressing pattern recognition receptors, including toll-like receptors (TLRs) and NOD-like receptors (NLRs), MPs can sense both danger-associated molecular patterns and pathogen-associated molecular patterns from damaged tissue and microbes, respectively (28). The trigger for activation of MPs in the CNS is unknown, but is thought to be a combination of genetic susceptibility and environmental factors. M1-like MP polarization results in the release of pro-inflammatory cytokines, including tumor necrosis factor (TNF)-α and interleukin (IL)-1β; chemokines, such as monocyte chemoattractant proteins and reactive oxygen species (ROS), through increased nicotinamide adenine dinucleotide phosphate (NADPH) oxidase activity. All these factors contribute to demyelination, gliosis, and axonal loss, thus leading to irreversible tissue damage (29). Pro-inflammatory cytokines indirectly damage neurons and oligodendrocytes (ODCs) through sensitization of axons to glutamate excitotoxicity (30, 31). Chemokines promote the recruitment of innate immune cells and T cells to the site of ROS production in the CNS, causing mitochondrial dysfunction of neuronal cells and an increased energy demand due to inefficient nerve conductance, which can result in axonal damage and neuronal death (32, 33).

The role and function of microglia in progressive MS still remains a matter of debate, especially considering the intrinsic plastic nature of these cells (34–36). Classifying the different phenotypes of microglia *in vivo* (i.e., applying to microglia the *old* M1-like vs. M2-like classification of macrophages) has proven challenging. Unlike macrophages, microglial cells are not professional antigen-presenting cells, but they quickly increase the expression of MHC class-I and -II complexes in response to injuries and/or local inflammation. Specifically during brain inflammation, T cells crossing the blood–brain barrier, directly interact with microglia to recognize antigens, and ultimately mediate their skew toward M1-like activation (37). Activated microglia release the Th1-like pro-inflammatory cytokine interferon (IFN)-γ, which might induce their own polarization via IFNGR, trough an autocrine loop (38). This potentially vicious cycle typical of progressive MS, in which microglia contribute to the self-propagation of neuroinflammation, is likely to be determined also by a failure in the M2-like responses in a microenvironment dominated by Th1/Th17 cytokines (39).

In the context of brain repair, the activation of microglia is also necessary for clearing debris and, more importantly, to support the remyelination of damaged axons (40–42). In Cuprizone-fed mice, an animal model of demyelination/remyelination, microglia sustain remyelination with a durable effect involving (i) the phagocytosis of myelin debris and apoptotic cells during demyelination and (ii) the expression of a repertoire of cytokines and chemokines that include insulin-like growth factor (IFG)-1, platelet-derived growth factor (PDGF)-α, and transforming growth factor (TGF)-β, which ultimately mediate the recruitment of oligodendrocyte precursor cells (OPCs) and their differentiation into mature functional ODCs (43). Indeed, remyelination of damaged axons is a process that can be driven by M2-like MPs (21). In mice with lysolecithin-induced focal demyelination of the corpus callosum, a switch from M1-like to M2-like phenotype is described for both microglia and peripheral macrophages. This M2-like polarization takes place as early as remyelination begins, and it is indispensable to promote OPC differentiation. Interestingly, M2-like MPs also produce neurotropic and growth factors, such as the TGF-β superfamily member activin-A, a key signaling intermediate for ODC function, thus contributing to the remyelination process (21). During progressive MS, this remyelination-supportive microglia phenotype may be impaired, thus preventing proper repair. Interestingly, the same failure of M2-like microglia seems to occur also in other neurodegenerative diseases characterized by chronic MP activation in the CNS, such as Alzheimer’s disease (44, 45).

## Metabolic Reprograming of MPs in MS

**Figure 1:**
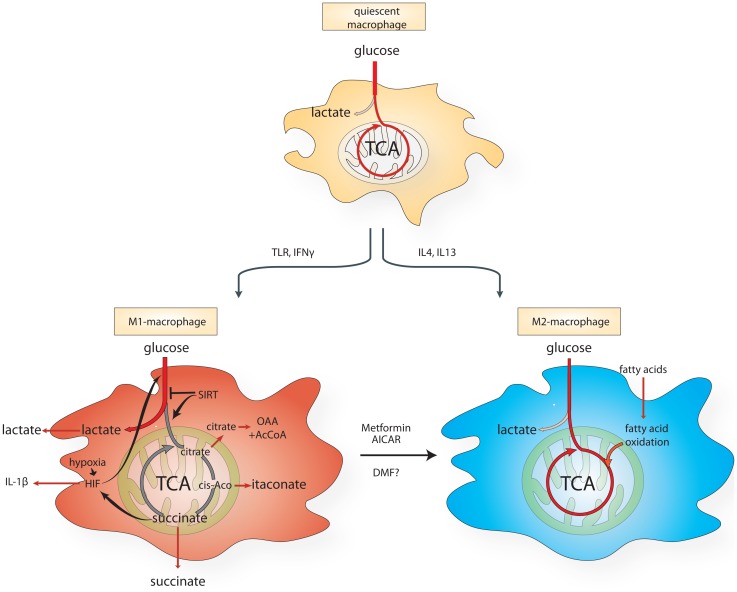
**Schematic diagram of the metabolic reprograming of macrophages undergoing M1- vs. M2-like polarization**. AICAR, aminoimidazole carboxamide ribonucleotide; cis-aco, cis-aconitase; DMF, dimethyl fumarate; HIF, hypoxia-inducible factor; IL, interleukin; IFN-γ, interferon-γ; TCA, citric acid cycle; TLR, Toll-like receptor.

Already in the 1980s, macrophages were known to undergo profound metabolic changes upon activation (46). More recent evidence corroborated these findings. While quiescent macrophages predominantly use mitochondrial respiration to generate energy, lipopolysaccharide (LPS)-activated macrophages switch their metabolism from oxidative phosphorylation to glycolysis (47, 48) (Figure 1). This metabolic switch, also known in cancer biology as the *Warburg effect* (49), is a key feature of the M1 macrophages. By contrast, mouse IL-4-stimulated MPs activate mitochondrial metabolism, fatty acid uptake, and fatty acid oxidation (50). These observations indicate that the switch between glycolytic or oxidative metabolism could play a role in macrophage polarization (Figure 1). However, the relevance of some of these metabolic features in human macrophages is still debated and more work is required to clarify the differences between mouse and human models.

### Hypoxia controls the M1 polarization of MPs

The switch toward aerobic glycolysis in activated macrophages is orchestrated – at least in part – by the transcription factor hypoxia-inducible factor (HIF) 1. HIFs are heterodimeric proteins composed of a constitutively expressed beta subunit and an oxygen-dependent alpha subunit (HIF-1α). In the presence of oxygen, the oxoglutarate-dependent prolyl hydroxylases (PHDs) hydroxylate HIF-1α, producing succinate and carbon dioxide. Once hydroxylated, HIF-1α is targeted for proteasomal degradation. When oxygen becomes limiting, PHDs are inhibited, leading to HIF-1α stabilization and to the activation of its transcriptional program, which drives the expression of numerous glycolytic enzymes and of the pro-inflammatory cytokine IL-1β (47, 51). Sites of inflammation in MS are often hypoxic and acidic (33, 52, 53) and, accordingly, tissue from MS lesions exhibits hypoxic markers, including increased expression of glucose transporters (GLUTs) and monocarboxylate transporters (MCT), compared with healthy patients (54), most likely due to increased glycolysis of activated MPs. The increased avidity for glucose by MS lesions can be exploited for diagnostic purposes, since these lesions uptake substantial amounts of the glucose analog [^18^F]-fluorodeoxyglucose, which can be visualized by positron emission tomography during neuroimaging exams (55). In line with these findings, tissue hypoxia and the corresponding increased expression of HIF-1α develops rapidly in response to inflammation in white and gray matter of animals subject to EAE. Levels of hypoxia correlate with neurological defects and the reintroduction of oxygen restores function in EAE mice within an hour of treatment, lasting up to 1 week (56). Interestingly, MPs and microglia from MS patients show different levels of HIF-1α, with MPs increasing HIF-1α expression compared to microglia (11, 23, 57). These observations suggest that MPs, rather than microglia, could be actively involved in the glycolytic switch observed in MS inflammation sites.

### Mitochondrial metabolites and regulation of MPs function

Changes in mitochondrial metabolism have important implications for activated macrophages, beyond cellular energetics. It has been recently shown that succinate, which accumulates in LPS-activated macrophages, impairs the enzymatic activity of PHDs by product-inhibition, leading to HIF-1α stabilization even in the presence of oxygen, a phenomenon known as *pseudohypoxia* (47). Importantly, manipulating succinate levels in macrophages *in vitro* can both stabilize HIF-1α as well as drive IL-1β expression (47). Furthermore, the inhibition of succinate dehydrogenase (SDH), the enzyme that converts succinate to fumarate, with diethylbutylmalonate increases intracellular succinate in macrophages and exacerbates the production of LPS-induced IL-1β (47). Interestingly, SDH is less active in the microglia of rats with EAE (58), underlining a possible deregulation of the enzyme in this experimental model of MS.

As well as acting intracellularly, succinate can also be released in the extracellular milieu, where it has been shown to act as a hormone-like molecule. High concentrations of succinate have been detected in the plasma of patients with peritonitis, in the urine and plasma of diabetic and metabolic disease rodent models (59, 60), and in the synovial fluid of patients with rheumatoid arthritis (61). Interestingly, succinate accumulation is induced as a response to ischemia in several tissues including the brain (62), thus suggesting that several factors could contribute to increase succinate levels in the microenvironment of MS lesions. Succinate has been shown to signal via the G-protein coupled succinate receptor 1 (SUCNR1), a protein highly expressed on a variety of tissues, including the spleen (63) and in immune cells (64). The activation of SUCNR1 by succinate synergizes with TLRs on dendritic cells and is required for enhanced antigen-presenting function of these cells (64). Therefore, blocking succinate receptor on MPs using the highly specific and potent SUCNR1 antagonist (65) could prove to be effective for the treatment of progressive MS.

Two other mitochondrial TCA metabolites, itaconic acid (ITA) and citrate have been shown to be involved in macrophage inflammatory pathways (Figure 1). ITA is induced and secreted by macrophages upon LPS and IFN-γ stimulation (66) and it inhibits the growth of bacteria that express isocitrate lyase, such as *Salmonella enterica* and *Mycobacterium tuberculosis*. Immunoresponsive gene 1 (Irg1) protein is the enzyme responsible for the production of ITA in mammalian cells. Gene silencing of *Irg1* in macrophages caused a substantial reduction in antimicrobial activity during bacterial infections (67).

Citrate is another important mediator of LPS-induced signaling in macrophages. Although produced exclusively in the mitochondria, citrate can be exported into the cytosol by the mitochondrial citrate carrier (CIC), and converted to oxaloacetate and acetyl CoA by the enzyme ATP-citrate lyase (ACLY). Interestingly, upon LPS, CIC expression levels increase and its inhibition (68) or the silencing of ACLY (69) was shown to block LPS-induced nitric oxide (NO), ROS, and prostaglandin production in macrophages, consistent with a role of cytosolic citrate as important precursor for these molecules.

In summary, the evidence reported above suggests that the mitochondrial dysfunction observed in MS lesions can lead to imbalance of several mitochondrial metabolites that, beyond being mere intermediates in energy metabolism, can directly influence the immunological function of different cell types involved in MS inflammation. Further understanding of the regulation of these metabolites will be important for the identification of targets to modulate MP metabolism.

## Metabolic Re-Education of MPs in MS

Given the relevance of metabolism in the activation and polarization of MPs, it has been proposed that an M1-to-M2 transition can be achieved by altering cell metabolism. For instance, it has been proposed that activating the key metabolic regulator AMP-activated kinase (AMPk) in MPs would enhance an M2-like phenotype by pushing oxidative metabolism. Indeed, metformin and 5-aminoimidazole-4-carboxamide-1-β-4-ribofuranoside (AICAR), well-established AMPk activators can attenuate progression of chronic EAE in mice by inhibiting macrophage infiltration into the CNS (70) and modulating the endothelial–macrophage interaction (71). Interestingly, AMPk-null mice have more severe EAE through an increase in macrophage infiltration to the spinal cord (72).

Another recently proposed metabolic strategy to polarize MPs to an M2-phenotype is the modulation of sirtuins, a family of seven NAD-dependent lysine deacetylases involved in a plethora of cellular processes, including metabolic homeostasis (73–75). Among the sirtuins, SIRT1, SIRT3, and SIRT6 play a key role in the regulation of cellular metabolism. For instance, SIRT3 regulates the enzymatic activity of SDH (76) and both SIRT1 and SIRT6 coordinate a switch from glycolysis to fatty acid oxidation in macrophages (77). Moreover, nicotinamide phosphoribosyltransferase (NAMPT), an important enzyme for NAD^+^ biosynthesis and sirtuins function, is required for the inhibition of prolonged macrophage activation via TLR4, indicating that sirtuins can act as anti-inflammatory factors during physiological response to pathogens (78). Therefore, sirtuins activity in MPs could favor an anti-inflammatory M2-like phenotype, by re-educating intermediary metabolism of these cells.

In conclusion, the manipulation of metabolic pathways is a tempting strategy to regulate MPs function in MS. However, using small molecules to regulate ubiquitous enzyme and metabolite levels may be cumbersome as metabolic pathways are crucial for normal cell function and energy production. Therefore, more selective strategies to target MP metabolism are required to regulate inflammation without impacting on metabolism of other tissue.

## Dimethyl Fumarate as Regulator of Mononuclear Phagocyte Metabolism in MS

A current therapy for relapsing MS is oral dimethyl fumarate (DMF; *Tecfidera*), a methyl ester of fumaric acid that is rapidly hydrolyzed to its active metabolite monomethyl fumarate (MMF), and shown to have a significant effect on relapse rate and time to progression in phase III clinical trials of MS (79, 80). Fumaric acid has been long licensed for the treatment of psoriasis (81), and progressive multifocal leukoencephalopathy, a rare potentially fatal neurologic disease caused by reactivation of JC virus infection, has been reported in rare cases (82, 83).

The mechanisms of action of DMF are still under investigation. DMF interacts with immune cells in the circulation and promotes a shift in cytokine production from a Th1-like (pro-inflammatory) to Th2-like (anti-inflammatory) pattern. Despite being approved for T-cell-mediated relapsing MS only, DMF has been shown to affect MPs *in vivo* in animal disease models. During the acute phase of EAE, Mac-3-positive microglia and macrophages are significantly reduced in DMF-treated mice (84). *In vitro* studies show that DMF can shift MPs from an M1-like to an M2-like phenotype. Evidence for the anti-inflammatory properties of DMF are shown in human PBMCs treated with either IFN-γ or LPS where the expression of the chemokines CXCL8, CXCL9, and CXCL10 are dose-dependently inhibited by DMF (85). In addition, application of MMF to MPs results in increased expression of the anti-inflammatory cytokines IL-4, IL-5, IL-10, and IL-1RA (86). In human macrophages, DMF and MMF block NF-κB activity by inhibiting its nuclear translocation and DNA binding in response to TNF-α and also reduce TNF-α (87). Furthermore, DMF and MMF suppress CCL2-induced chemotaxis of human MPs (87). These data suggest that this block in chemotaxis would result in decreased infiltration of MPs into the CNS across endothelial surfaces. It has also been proposed that DMF may play a role in CNS oxidative stress by activating the nuclear factor (erythroid-derived 2)-related factor-2 (Nrf2), a transcription factor with antioxidant properties (88, 89). LPS-induced NO, TNF-α, IL-1β, and IL-6 expression in microglia cells is reduced by pre-treatment with DMF, possibly through activation of Nrf2 pathway (90). However, this effect has not been demonstrated *in vivo* in EAE mice (91). In addition, fumaric acid esters induce up-regulation of superoxide in monocytes, which is indicative of a pro-inflammatory response (92). Although the exact mechanisms of action of DMF are not fully understood and still controversial, we postulate that, since the active form of DMF is fumarate, a TCA cycle metabolite, this molecule may act also by altering the metabolism of MPs and favoring an M2-phenotype. However, more data is required to validate this hypothesis.

## Summary

The link between metabolism and inflammation has become a hot topic over the past 5 years. The metabolic state of MPs is now thought to affect their *inflammatory status*. Understanding the changes in metabolism that occur in inflammatory and autoimmune diseases is crucial to interpret disease pathogenesis and identify novel therapies for progressive MS. Here, we provided evidence to show that targeting specific metabolic processes in MPs to regulate their inflammatory state might be used as an MS therapy.

## Conflict of Interest Statement

The authors declare that the research was conducted in the absence of any commercial or financial relationships that could be construed as a potential conflict of interest.
